# Achieving Ultra-Wideband and Elevated Temperature Electromagnetic Wave Absorption via Constructing Lightweight Porous Rigid Structure

**DOI:** 10.1007/s40820-022-00904-7

**Published:** 2022-08-23

**Authors:** Zibao Jiao, Wenjun Huyan, Feng Yang, Junru Yao, Ruiyang Tan, Ping Chen, Xuewei Tao, Zhengjun Yao, Jintang Zhou, Peijiang Liu

**Affiliations:** 1 College of Materials Science and Technology, Nanjing University of Aeronautics and Astronautics, Nanjing, 211100 People’s Republic of China; 2 Key Laboratory of Material Preparation and Protection for Harsh Environment (Nanjing University of Aeronautics and Astronautics), Ministry of Industry and Information Technology, Nanjing, 211100 People’s Republic of China; 3 School of Electronic Science and Engineering, Nanjing University, Nanjing, 210023 People’s Republic of China; 4 School of Materials Science and Engineering, Nanjing Institute of Technology, Nanjing, 211167 People’s Republic of China

**Keywords:** Porous structure, EM wave absorption, Mechanism

## Abstract

Realizing ultra-wideband absorption, desirable attenuation capability at high temperature and mechanical requirements for real-life applications remains a great challenge for microwave absorbing materials. Herein, we have constructed a porous carbon fiber/polymethacrylimide (CP) structure for acquiring promising microwave absorption performance and withstanding both elevated temperature and high strength in a low density. Given the ability of porous structure to induce desirable impedance matching and multiple reflection, the absorption bandwidth of CP composite can reach ultra-wideband absorption of 14 GHz at room temperature and even cover the whole X-band at 473 K. Additionally, the presence of imide ring group in polymethacrylimide and hard bubble wall endows the composite with excellent heat and compressive behaviors. Besides, the lightweight of the CP composite with a density of only 110 mg cm^−3^ coupled with high compressive strength of 1.05 MPa even at 453 K also satisfies the requirements in engineering applications. Compared with soft and compressible aerogel materials, we envision that the rigid porous foam absorbing material is particularly suitable for environmental extremes.

**Supplementary Information:**

The online version contains supplementary material available at 10.1007/s40820-022-00904-7.

## Highlights

- Constructing a porous carbon fiber/polymethacrylimide (CP) structure for acquiring promising electromagnetic absorption performance and withstanding both elevated temperature and high strength in a low density.
- The absorption bandwidth of CP composite can reach ultra-wideband absorption of 14 GHz at room temperature and even cover the whole X-band at 473 K.
- The lightweight of the CP composite with a density of only 110 mg cm^−3^ coupled with high compressive strength of 1.05 MPa even at 453 K.

**Supplementary Information:**

The online version contains supplementary material available at 10.1007/s40820-022-00904-7.

## Graphical abstract

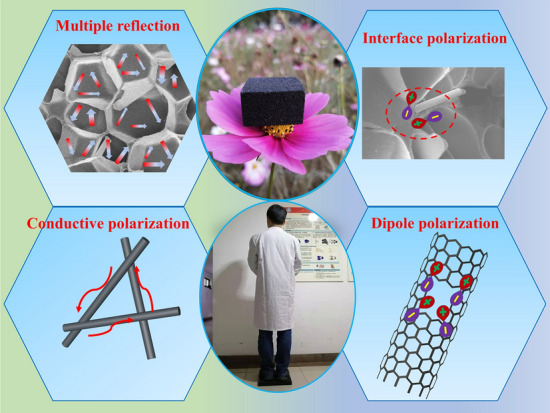

## Introduction

Electromagnetic (EM) wave absorbing materials can eliminate the EM pollution produced by EM equipment during operation and therefore offer important potential in the field of military stealth and 5G [1–8]. In the quest for better EM compatibility, the development trend of new electronic instruments is multiple and wide frequency band [9–13]. Thus, there is an urgency for high-quality microwave absorbing materials with wide band absorption to eliminate the excess EM pollution generated during equipment operation [14–18]. Previous research work mainly focused on the control of micromorphology and the optimal combination of dielectric and magnetic constitutions [19–26]. However, considering the frequently used range of 2–18 GHz, realizing an effective absorption bandwidth (EAB) of more than 10 GHz is still an unachievable challenge.

Researchers found that constructing porous structures is more conducive to microwave absorption (MA) promotion compared with using solid particles under the same amount [27–32]. Freeze–drying method is the most common way for constructing porous structures in terms of aerogel and foam sponge. For example, Liu et al. [33] reported a simple freeze–drying technology to prepare porous rGO aerogels with a wide EAB up to 7.47 GHz. Zhang and his colleges [34] prepared ultra-light graphene foam by hydrothermal method, and the EAB can reach amazing 12 GHz. It is well accepted that the impedance matching characteristic of porous structure is very close to air, thus most of EM waves propagate in the absorbent material rather than be reflected. Further, the porous structure also gives rise to multiple scattering and multiple reflection of EM wave, which is beneficial to the extension of transmission route and subsequent conversion of EM energy into heat energy. At some point, construction of a 3D conductive network through porous structure is the key to expanding perspectives on ultralight and high-performance broadband MA materials. However, the ultra-wide porous absorbing material is still facing two critical issues. First of all, such ultra-wideband absorbing performance is readily achieved at room temperature. Nevertheless, considering the various applicabilities toward a high-temperature (up to 473 K) or harsh environment, absorbing frequency band usually requires to cover a specified region such as but not limited to full band coverage in military X-band [35–37]. Secondly, the porous structure of aerogel is soft and compressible to some extent that is a lack of ability to withstand certain compression. When subjected to pressure, the interior structure and size of aerogel proceed to change, then resulting in an uncontrollable reversal of absorbing behavior. In view of these facts, it is necessary and urgent to explore a novel kind of porous foam with excellent EM attenuation, superior heat resistance, and great compression resistance both at room and high temperature.

The polymethacrylimide (PMI) foam features a rigid structure with temperature resistance up to 220 °C and close cell content of more than 90%. Moreover, its uniform cross-linked pore wall structure affords outstanding structural stability and excellent mechanical properties [38]. Without considering other aspects, PMI foam exhibits higher compressive strength and stiffness than other polymeric foams at the same density, not to mention its high heat resistance [39, 40]. These fascinating features of lightweight PMI foam meet the ever-increasing needs of aerospace for lightweight and high strength. Besides, employment of PMI greatly reduces the overall weight of structural parts and thus allows the simplicity of mechanical structure design. Relying on the high heat resistance (up to 453 K) and pressure resistance (about 0.6 MPa), the fabrication of PMI is therefore applicable to autoclave co-curing process, and mainly adopts acrylonitrile and acrylic acid as monomer [41]. Although utilization of acrylonitrile and methacrylic acid as the main monomer reduces the overall cost of PMI products, the reaction side-effect that is intense exotherms of polymerization and even explosion caused by the difference of reactivity ratios of the two monomers is inevitable. This results in incomplete reaction and considerable residual molecules. More importantly, the inorganic fillers, especially carbon-based materials, undergo degeneration or oxidation procedure, causing irreversible deterioration of functionality. Consequently, maintaining the processing reliability of PMI whilst retaining other beneficial functions of inorganic fillers is still an enormous challenge.

In this work, we have designed a kind of carbon fiber/PMI (CP) composite by mixing carbon fibers (CFs) to the monomers and subsequently conducting water-bath polymerization. Through precisely controlling the amounts of CF absorbents, rational modulation of EM properties can be readily gained, therefore allowing to realize significant EM attenuation capability. Ultra-wideband absorption can also be gained under thinner thickness by using a genetic algorithm and multilayer design. Meanwhile, the excellent heat resistance and compressive strength of CP composite allow the EM attenuation effectiveness to cover whole X-band at high temperature (473 K). Conceptually, broadening the EAB of EM absorbing foams with rigid porous structure no matter at room temperature or elevated temperature provides a new idea for the design of novel absorbing materials.

## Experimental Section

### Materials

Methyl acrylonitrile was purchased from Hunan Huateng Pharmaceutical Co., Ltd., China. Methacrylic acid, light magnesium oxide, methyl methacrylate, isopropanol, tert butyl methacrylate, azobisisobutyronitrile, benzoyl peroxide and white carbon black were purchased from Macklin. Short, cut CF was purchased from Shanghai Liso Composite Material Technology Co., Ltd., China.

### Synthesis of CP Composites with Different CF Contents

Methacrylic acid, methyl acrylonitrile, light magnesium oxide, methyl methacrylate, isopropanol, tert-butyl methacrylate, azobisisobutyronitrile and benzoyl peroxide were added to a three-neck bottle, stirred for 30 min to ensure that the entire system is uniform. Then added white carbon black and started to stir quickly for 30 min until the whole system becomes viscous. Then the short cut CF was added, and stirring was continued for 15 min. The above viscous liquid was poured into a mold and then reacted in a water bath at 50 °C for 4 days to obtain prepolymers. The prepolymers were freely foamed in a 240 °C oven for 1 h to obtain different contents of CP foams.

### Characterization and Measurement

The microstructures of CP composites were observed by scanning electron microscope (SEM, Hitachi, S4800). The changes in groups during synthesis were analyzed using an infrared spectrometer (Bruker, Vector-33). The crystal structure of the composites was evaluated by X-ray diffraction (XRD, Bruker, D8 Advance). An X-ray photoelectron spectroscope (XPS) was used to analyze the changes in chemical states of the structures (Thermo Scientific, ESCALAB 250XI). A thermogravimetric analyzer (TGA) was used to characterize the heat resistance of the material, and N_2_ atmosphere was used during the test (NETZSCH, STA409PC). The EM parameters of the CP composites at room temperature were tested using a vector network analyzer (CeYear, 3672B) via a coaxial method, and the test frequency band was 2–18 GHz. The sample size was 7.00 mm in outer diameter, 3.04 mm in inner diameter and 2 mm in thickness. The EM parameters at high temperature were tested using waveguide method in the range of 8.2–12.4 GHz, and the sample size was 22.8 × 10.1 × 2 mm^3^. The reflection loss curve of the multilayer structures was measured via space method, and the sample size was 300 × 300 × 30 mm^3^.

## Results and Discussion

### Analysis of Microstructure of CP Composites

**Fig. 1:**
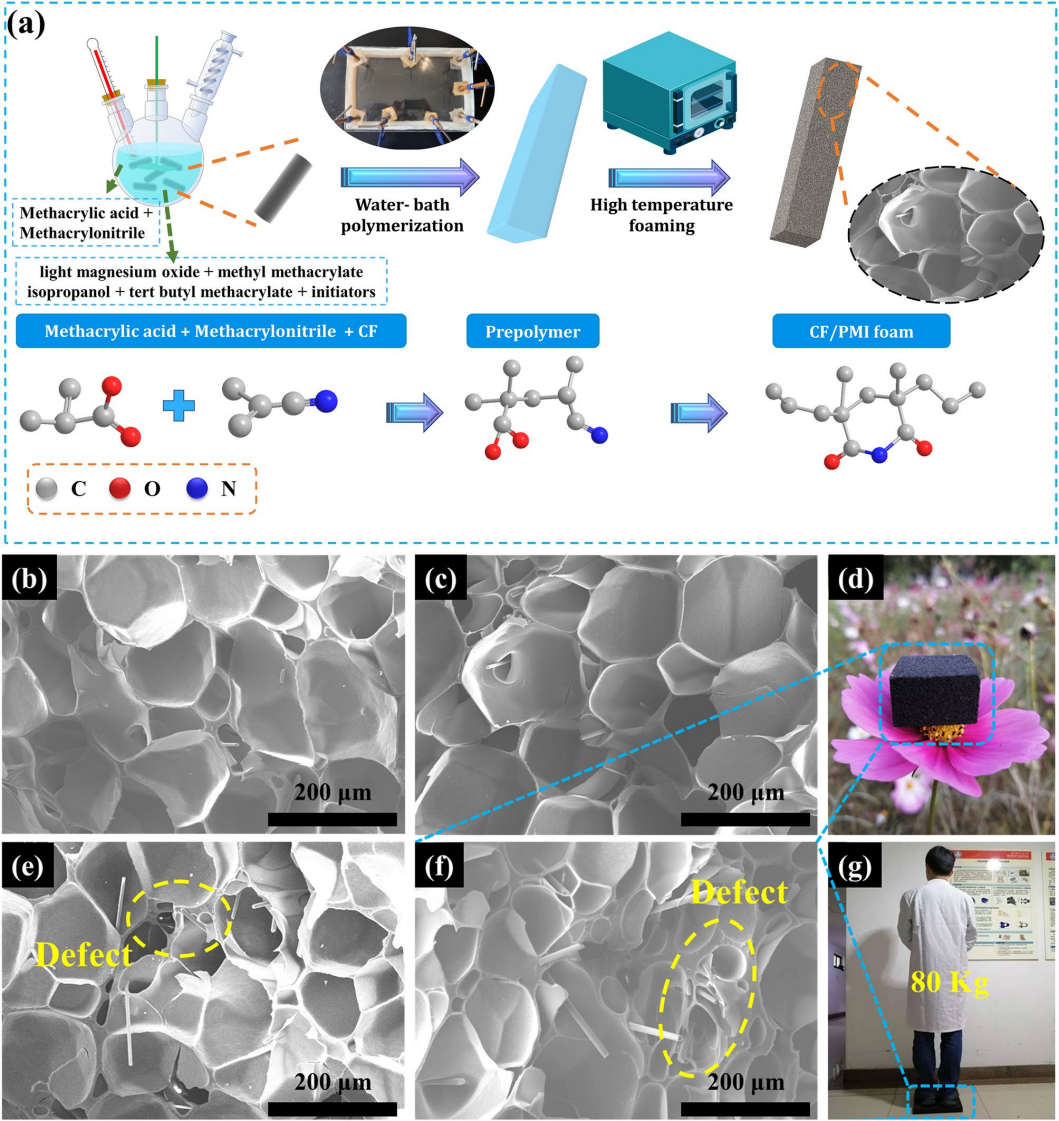
**a** Schematic illustration of the synthesis process for CP composites, SEM images of **b** CP-1, **c** CP-2, **e** CP-3, **f** CP-4, **d** photograph of CP-4 placed on the stamen and **g** photograph of a person stand on CP-4

The schematic diagram of the synthesis steps of CP is shown in Fig. 1a. The samples with mass fraction of CF in monomer (methacrylic acid and methacrylonitrile) of 0, 1, 2, 3, and 4% were denoted as CP-0, CP-1, CP-2, CP-3, and CP-4, respectively. After water bath treatment, methacrylic acid and methacrylonitrile polymerized into long-chain prepolymer. Afterward, the adjacent carboxyl group (–COOH) reacted with the cyanide group (–CN) to afford the heat-resistant imide ring during the foaming process. Fourier transform infrared spectroscopy (FT-IR) spectra illustrate the formation of imide ring structure (Fig. S1b), in which the peaks at 2239 and 1699 cm^−1^ are corresponding to the stretching vibrations of carbonyl group (–C=O) and cyanide group (–CN), respectively, and the peak at 1223 cm^−1^ relates to the complete vibration peak of C–N bond.

The morphologies of samples are presented in Fig. 1b–g, the order of which follows the above species of various CF contents. All CP composites exhibit a typical closed porous structure, whilst CF uniformly and evenly disperses in PMI interior. Excellent impedance matching is the first important step in EM absorption so that incident waves can unimpededly transmit on the absorber surface, which facilitates further EM attenuation. The porous structures of the CP composites offering substantial air are conducive to the impedance matching, so that substantial EM waves can enter the materials. At the same time, as the EM wave arrives the interior of materials, the porous structure will stimulate multiple scattering that is also advantageous to consume EM waves. When the amount of CF is less than 2 wt%, the size of bubble structures is relatively uniform and about 150–200 µm. However, as the content of CF increases, some defects and nonfoaming phenomena appear in the bubbles. Two reasons may be put forward: On the one hand, with the increase in CF, a few of unwanted agglomerations generate owing to van der Waals force and settlement effect during foaming process, which minimize the interface energy and thus hinders the solidification and foaming process. On the other hand, excessive amounts of CFs will break the bubble hole during the foaming process and further suppress cell expansion, as illustrated in Fig. 1e–f. These unpleasant defects and nonfoaming phenomena are unfavorable to either EM absorption or mechanical robustness. Therefore, the amount of CF was not further increased in this work. Owing to the poriferous and rigid structures, CP composites show a lightweight and high-strength character, as shown in Fig. 1d, g. The density of CP-4 is evaluated to be only 110 mg cm^−3^, and such a small density allows it to be placed on the stamen without damaging. In particular, it can also withstand the pressure of 80 kg without generating indentation. This lightweight and high-strength structure indicates a high application value in the aviation and aerospace fields.

In addition, the CP composites show excellent heat resistance. As can be seen from Fig. S1a, there is almost no thermal weight loss before 360 °C, an indicative of good thermal stability of the CP composites. This excellent heat resistance enables their application in the field of high-temperature MA. As the amount of CF increases, the heat resistance of the CP composites gradually increases, and the thermal deformation temperature of CP-4 remarkably improves and reaches 371.7 °C. Such excellent heat resistance is mainly derived from the imide ring groups on the molecular chain of PMI, which is of powerful help to the rigidity of the molecular chain. This strategy toward the improvement of the MA performance by constructing a porous structure does not change the chemical group, crystal structure and chemical state of the material (Fig. S1b–d).

### Analysis of Microwave Absorbing Properties of Single Layer CP Composites

EM parameters of the material have been measured via a coaxial method, and the relevant EM wave absorption performance was calculated in accordance with transmission line theory. The *R*_L_ value of the reflection loss can be expressed by the following formula:

1 $$ R_{\text{L}} = 20 {\text{log}}\left| \Gamma \right| = 20{\text{ log}}\left| {\frac{{Z_{{{\text{in}}}} - 1}}{{Z_{{{\text{in}}}} + 1}}} \right| $$

2 $$ Z_{{{\text{in}}}} = \sqrt {\frac{{\mu_{r} }}{{\varepsilon_{r} }}} \tan h\left[ {j\left( {\frac{2\pi fd}{c}} \right)\sqrt {\mu_{r} \varepsilon_{r} } } \right] $$

where, *R*_L_ is the reflection loss (dB), *Z*_in_ is the normalized input impedance, ε_*r*_ is the complex dielectric constant, μ_*r*_ is the complex permeability, *f* is the frequency of EM wave, *d* is the thickness of absorbing material, and c is the propagation speed of EM wave in vacuum.

**Fig. 2:**
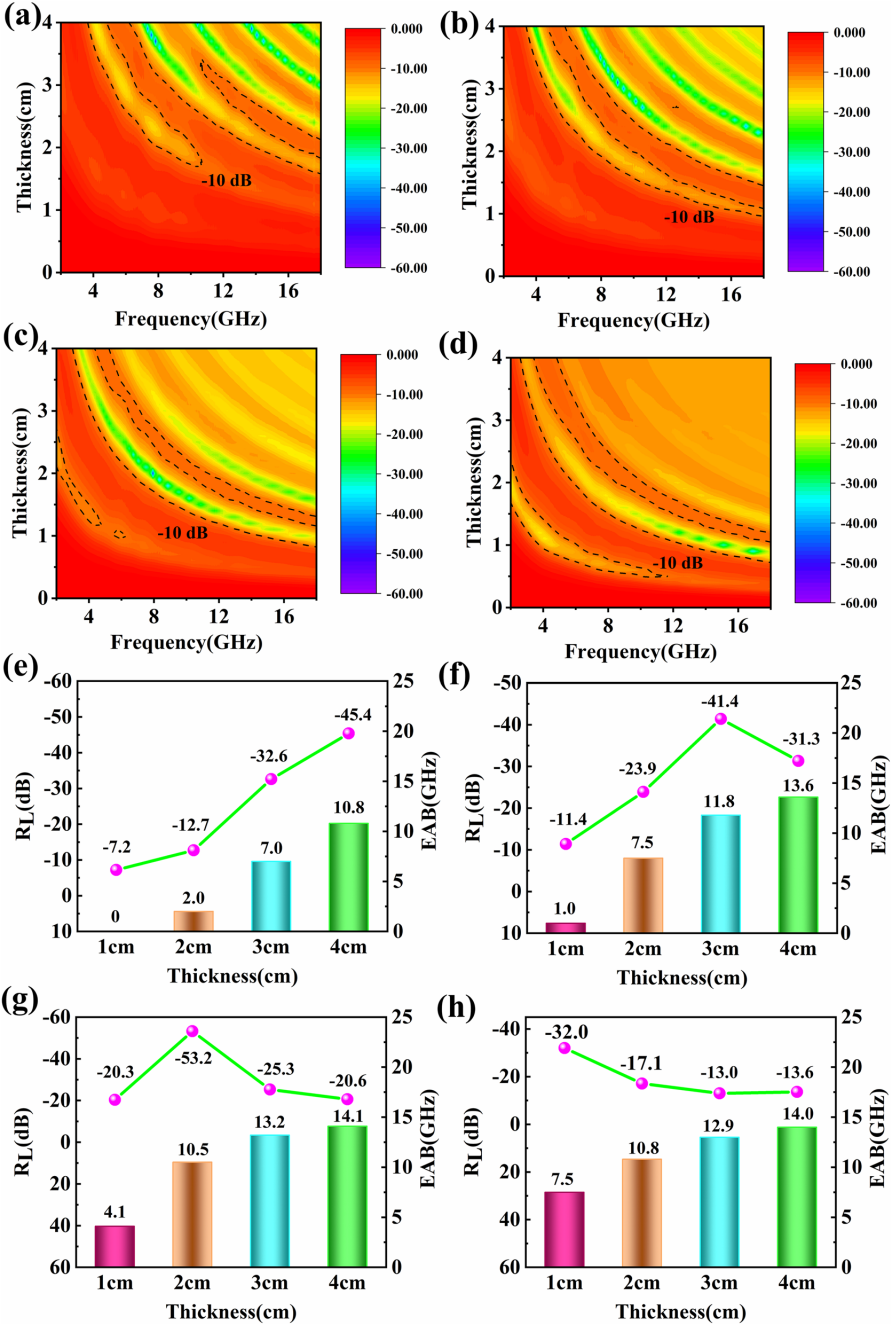
Two-dimensional *R*_L_ map of **a** CP-1, **b** CP-2, **c** CP-3, **d** CP-4, maximum reflection loss value and EAB at different thicknesses of **e** CP-1, **f** CP-2, **g** CP-3, **h** CP-4

The reflection losses at different frequencies and thicknesses are clearly shown in a two-dimensional contour map (Fig. 2a–d). The smaller the value of *R*_L_ is, the more EM wave energy is consumed. We regard −10 dB as the boundary to indicate the effective EM wave absorption. The frequency range with *RL* less than −10 dB is defined as the EAB, which is marked with dotted line in Fig. 2a–d. The peak of maximum *RL* value moving toward a lower frequency with the increase in thickness can be explained by *λ*/4 equation:

3 $$ d = \frac{{\lambda_{0} }}{4} \cdot \frac{1}{{\sqrt {\varepsilon_{{\text{r}}} \mu_{{\text{r}}} } }}\left( {2l - 1} \right) = \frac{c}{{4 \cdot f_{{\text{m}}} }} \cdot \frac{1}{{\sqrt {\varepsilon_{{\text{r}}} \mu_{{\text{r}}} } }}\left( {2l - 1} \right) $$

where, *d* is the thickness of the absorbing material at the maximum *R*_L_ value, λ is the wavelength and *f*_m_ is the frequency. It is clear that the thickness, frequency and electromagnetic parameters have relevance on the maximum *R*_L_ value. Because no magnetic material is used in this work, μ_r_ remains unchanged, ε_r_ increases with the amount of CF increases (Fig. S3). In addition, the *d* and *f*_m_ are the thickness and frequency of the absorbing material at the maximum RL value, while *c* is constant. Under the same thickness, the *f*_m_ is directly proportion to the square root of ε_r_. Accordingly, ε_r_ increases with the amount of CF increases, the RL_min_ value shifts to lower frequencies. The detailed EM absorption data of all samples are summarized in Fig. 2a–f. Undoubtedly, the CP composites show excellent MA properties. Even though the amount of CF is extremely low, an EAB greater than 10 GHz is achieved. More precisely, when the amount of CF is further increased, the EAB of CP-3 and CP-4 can reach as high as 14 GHz. The absorbing property of the composite material basically improves with the increase in CF under the same thickness. These findings suggest that the excellent absorbing performance is attributed to the synergetic behaviors of multiple mechanisms including the suited impedance matching, multiple reflections of porous structure, great polarization loss between components, and conductive loss of CF itself.

**Fig. 3:**
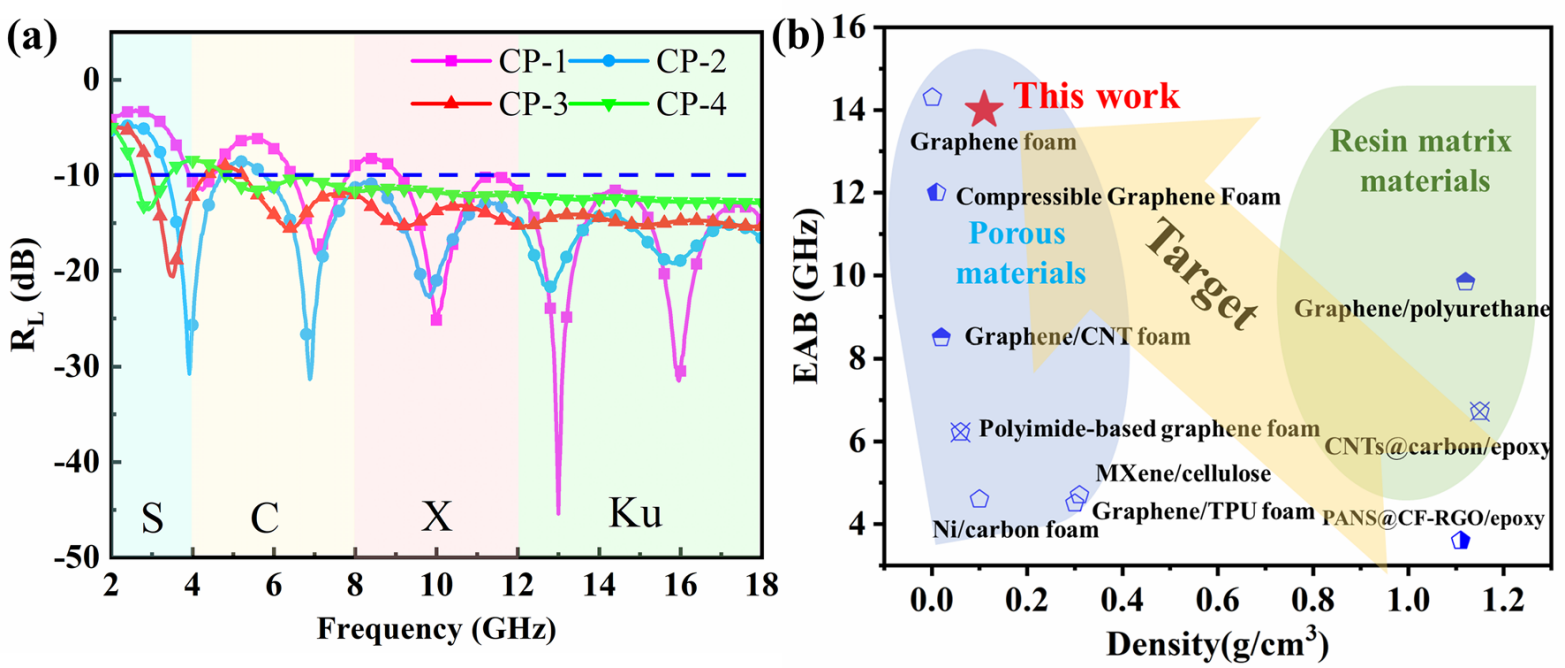
**a** *R*_L_ curves of CP composites with the thickness of 4 cm, **b** comparison with the MA properties of other absorbers reported previously

Aiming at elaborating the advancement and application of the present absorbing materials, *R*_L_ curves and the comparison of CP composites with other absorbers reported previously are exhibited in Fig. 3. The MA capacity of the CP-3 and CP-4 composites based on porous structure can cover the whole X and Ku bands and most of the C-band, suggesting a distinguished EAB with the 87.5% test range. In view of that, the CP composites fully exhibit advancement and practicability toward ultralight and ultra-wideband absorbing application compared with the porous structures [34, 42–46] and resin matrix composites [47–49] reported previously (Fig. 3b).

The *λ*/4 equation corresponds to interference in electromagnetic wave consumption. In the transmission line calculation model, the absorbing material is coated on the metal base plate by default. When the incident electromagnetic wave perpendicularly penetrates into the absorbing material and reaches the metal base plate, the corresponding reflection phenomenon will occur. During the period, interference behavior is present as the reflected electromagnetic wave encounters the incident electromagnetic wave, and the thickness *d* should satisfy Formula (3). With the increase in material thickness, multiple interference absorption peaks are identified. As shown in Fig. S2, the peaks of the reflection loss curves of CP composites at the thickness of 4 cm located on the *λ*/4 curves, indicating the well fit with the *λ*/4 model [50, 51]. The interference absorption peak is symbolized by sharp and strong absorption. With the increase in carbon fiber, these kinds of sharp peaks decrease, especially for CP-3 and CP-4, mainly because of the interference behavior and their own attenuation ability. The EM loss capacity of CP composite with the increase in carbon fiber dosage is exhibited in Fig. S4b. The loss capability enhancement validates the fact that enormous electromagnetic waves entering the material are basically consumed rather than reflected. As a result, interference will not occur. The reflection loss curve thus becomes relatively smooth with almost no sharp peaks [52]. This phenomenon is more prominent at high frequencies since EM wave with a shorter wavelength at high-frequency region is preferable to be consumed under the same material thickness. There still exists the partial interference after the electromagnetic wave which is not completely consumed at low frequency is reflected back. Thus, the reason for the degradation of the RL_min_ of CP-4 is that the reflection loss is mainly due to its own loss capacity of electromagnetic waves.

The absorbing properties of the absorbers heavily depend on their own EM parameters. To deeply investigate the source of absorbing properties, the EM parameters are analyzed. Given that the magnetic ingredients are not used in composite materials, only the dielectric behavior is studied when discussing the EM parameters. Accordingly, only polarization and conductive losses were present in the CP composites. With the increase in CF content, both the real part and the imaginary part of dielectric parameters show a rising trend (Fig. S2). The dielectric real part increases from 2.2 to 4.2, and the dielectric imaginary portion rises from 0.4 to 1.8, respectively. The protrusion of the imaginary part of the dielectric curve indicates the existence of multiple polarization relaxation processes. With the increase in the amount of CF, the heterogeneous interface between the carbon fiber and the PMI foam increases, which shows the increase in the imaginary part of the dielectric [53, 54]. In Fig. S3b, the protrusion of CP-4 composite material at 3–4 GHz is more obvious than that of other composite materials. In general, the dielectric loss positive cut value represents the dielectric loss capability of the absorbing material. In Fig. S3c, the maximum dielectric loss tangent of CP-4 can reach about 0.5, which implies the super dielectric loss ability of CP-4 composite. In order to provide comprehensive views of the dielectric loss, the imaginary part of dielectric (ε″) is divided into polarization loss (ε_p_″) and conductive loss (ε_c_″), which can be calculated using the following formula in accordance with Debye relaxation theory:

4 $$ \varepsilon_{c} ^{\prime\prime} = \frac{{\upsigma }}{{{\upomega }\varepsilon_{0} }} $$

5 $$ \varepsilon_{p}^{^{\prime\prime}} = \frac{{\varepsilon_{s} - \varepsilon_{\infty } }}{{1 + {\upomega }^{2} {\uptau }^{2} }}\omega \tau = {{\varepsilon^{\prime\prime}}} - \varepsilon_{c} ^{\prime\prime} $$

where* σ* is the electrical conductivity, $\upomega$ is the angular frequency,* ε*_0_ is the permittivity of vacuum, and τ is the period of relaxation. CP-4 is analyzed in accordance with the above equation, as shown in Fig. S3d. With the increase in frequency,* ε*_c_″ decreases rapidly, whereas* ε*_p_″ increases gradually. Concretely,* ε*_c_″ is dominant in the low frequency domain (2–3.8 GHz), whereas ε_p_″ is dominant in the high frequency domain (3.8–18 GHz). This features evidences that when the frequency is relatively high, the steering of the dipole cannot keep up with the EM change, resulting in the relaxation process. In the EM field, as the direction in the EM field changes constantly, the positive and negative charges of the electron cloud are constantly separated, and thus polarization loss is generated.

When the EM wave is incident on the surface of the absorbent material, a portion of the EM wave enters the inside of the material, and other EM waves are reflected in the free space. The reflected EM wave is unable to be consumed, so the primary condition for the consumption of EM wave is to facilitate as much EM wave into the material as possible. The index representing the EM wave entering into the material is normalized input impedance. The larger the value is, the better the impedance matching characteristic of material is. Whether the EM wave entering the absorbing material can be consumed depends on the attenuation coefficient of the material itself. The normalized wave impedance matching coefficient* Z* and attenuation coefficient α can be expressed by the following formula:

6 $$ Z = \frac{{Z_{1} }}{{Z_{0} }} = \left| {\sqrt {\frac{{\mu_{r} }}{{\varepsilon_{r} }}} } \right| $$

7 $$ {\upalpha } = \frac{\sqrt 2 \pi f}{c} \times \sqrt {\left( {\mu^{\prime\prime}\varepsilon^{\prime\prime} - \mu ^{\prime}\varepsilon ^{\prime\prime}} \right) + \sqrt {\left( {\mu^{\prime\prime}\varepsilon^{\prime\prime} - \mu ^{\prime}\varepsilon ^{\prime}} \right)^{2} + \left( {\mu^{\prime}\varepsilon^{\prime\prime} + \mu ^{\prime\prime}\varepsilon ^{\prime}} \right)^{2} } } $$

As mentioned above, the construction of a porous structure is conducive to improving the impedance matching characteristics. Even for the CP-4 composites with the worst impedance matching, most of the *Z* values are above 0.5 (Fig. S3a), which are significantly better than those of the common microwave absorber/paraffin composite. The strategy of improving the impedance matching of microwave absorbing materials by constructing porous structures is feasible and effective. Such excellent impedance matching characteristics enable ultrawide frequency absorption. Obviously, with the increase in the amount of CF, the attenuation ability of microwave absorbing materials to EM waves is enhanced. For CP-3 and CP-4 composites, their attenuation coefficients reach the maximum values of as high as 68 and 91, respectively (Fig. S3b). The cooperative role of impedance matching and attenuation capacity enables CP-3 and CP-4 to achieve ultrawide effective absorption of about 14 GHz.

### Multilayer Structure Design and Characterization

**Fig. 4:**
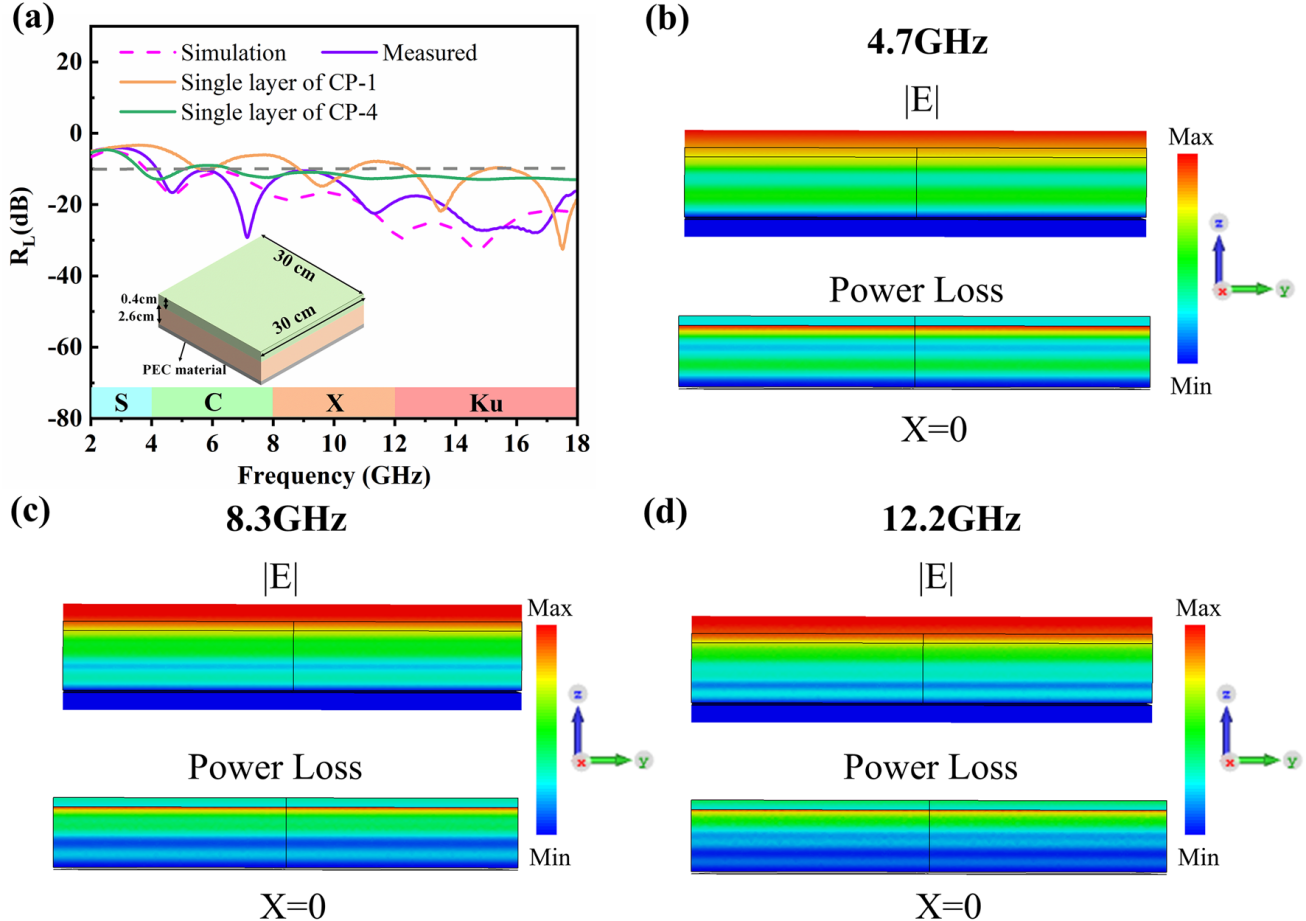
**a** Results of multilayer optimization design, simulation of electric field intensity and power loss at **b** 4.7 GHz, **c** 8.3 GHz and **d** 12.2 GHz

Through multilayer design optimization, the performance of each layer can be complementary, and the EAB can be further expanded at the same thickness. Alternatively, the total thickness of the multilayer material can be further reduced at certain absorbing properties. Genetic algorithm is the most effective method to design multilayer absorbing materials. In this study, EM parameters of each layer absorbing material are regarded as database resources, the total thickness is limited to 3 cm, the number of layers of multiple layers is less than three layers, effective absorption in the range of 4–18 GHz is considered convergence conditions, and optimization is carried out using a genetic algorithm. When the CP-1 material with the thickness of 0.4 cm is used in the upper layer, and CP-4 material with the thickness of 2.6 cm is used in the lower layer, the optimized simulation results of the reflection loss suggest that an effective absorption of 4–18 GHz (14 GHz) is achieved, as shown in the purple dotted line in Fig. 4a. In comparison, the EABs of the single-layer CP-1 and CP-4 materials at the same thickness are only 7.0 and 12.9 GHz, respectively. We may deduce that the multilayer design can maximize the absorbing effect of the absorbent material, and excellent absorbing properties can be achieved at a thinner thickness. For comparison, the real MA performance of objective CP-4 is tested via space method. The sample integrated by the 30 × 30 × 0.4 cm^3^ CP-1 material and the 30 × 30 × 2.6 cm^3^ CP-4 material has been fabricated through the vacuum bag pressing process to fulfill the measure requirement. As shown in Fig. 4, the measured absorbing performance is basically consistent with the simulation result, and the position of absorption peak is slightly offset, which may be related to the size error of the material in the preparation process. To analyze the absorption mechanism of the multilayer structure, CST Microwave Studio is used to conduct in-depth research on the electric field intensity and power loss distribution of each absorption peak frequency point, as depicted in Fig. 4b–d. The maximum value of electric field intensity mainly concentrates at the first layer and between the two layers of the multilayer structure, which is caused by interference of multiple *λ/*4. From the power loss image, the power loss mainly concentrates on the surface of the second layer, where the EM wave energy is consumed in large quantities. As for the first layer, the power loss is relatively low, implying that the first layer is mainly a matching layer and more EM waves are allowed to enter the interior of the multilayer structure. Besides, as the frequency increases, the power loss becomes increasingly concentrated on the upper and surface of the second layer. This may be considered that the wavelength *λ* of EM waves becomes small as the frequency increases, and the EM wave can be consumed within a shorter transmission path after the EM wave enters the structure.

### Analysis of Absorbing Mechanism

**Fig. 5:**
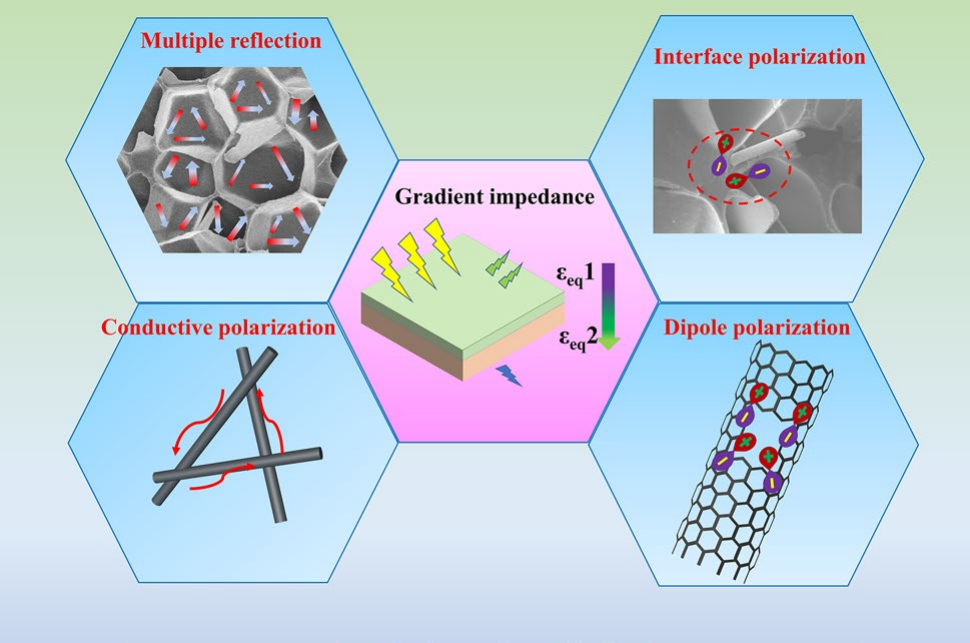
Schematic diagram of the absorbing mechanism of CP composite

Based on the above results discussions and simulation analysis, the schematic diagram of the absorbing mechanism of CP composite is shown in Fig. 5. On the macroscale, the impedance gradient structure is conducive to enhanced impedance matching. The dielectric parameters of materials with low dielectric coefficient are closer to those of air, which can be used as a matching layer to facilitate EM waves to enter the structure. The porous structure of CP composites, which contain a large amount of air, rendering a better impedance matching characteristic compared with conventional absorbing materials [55, 56]. This strategy to improve the MA performance by constructing a porous structure is an important first step to broaden EAB. Simultaneously, multiple reflections occur when EM waves enter the porous structure, which improve the travel distance of EM waves in the structure, resulting the EM waves being consumed in a shorter time. From the microscopic point of view, the consumption of EM wave mainly comes from the conductive loss, the polarization relaxation of dipole, the polarization relaxation of interface and the defect induced polarization. Under the action of an external electric field, electrons of CF will move and cause weak current, resulting in conductive loss. The 3D network forms a local conductive network, which is highly advantageous for the formation of conductive loss. Additionally, the existence of polar functional groups such as carbonyl radical contributes to the dipoles. Under the action of an alternating electric field, the rotation of dipoles cannot keep up with the speed of EM, which leads to dipole polarization. Dipole polarization relaxation basically occurs in a high-frequency range, which is mainly caused by the faster turning speed of the electric field at the high frequency. Dipole polarization consumes electromagnetic waves in the form of thermal motion of the dipole under the action of an applied alternating electric field. In addition, CF and PMI are two components with different dielectric properties. When additional EM fields are applied, interface polarization occurs between the interfaces of the two components. These two substances are structured capacitor-like and collect positive and negative charges at the ends of the capacitor. When the direction of the applied EM field changes constantly, the positive and negative charges between the two plates also change constantly, thus forming interface polarization. More importantly, grain boundary as a kind of planar defect is commonly found in absorbers with multiple components. The existence of defect sites on interfaces of different components for accommodating their lattice mismatch. In this regard, both interfacial polarization and defect-induced polarization can lead to EM wave loss.

### Analysis of High Temperature Mechanical and Microwave Absorbing Properties

**Fig. 6:**
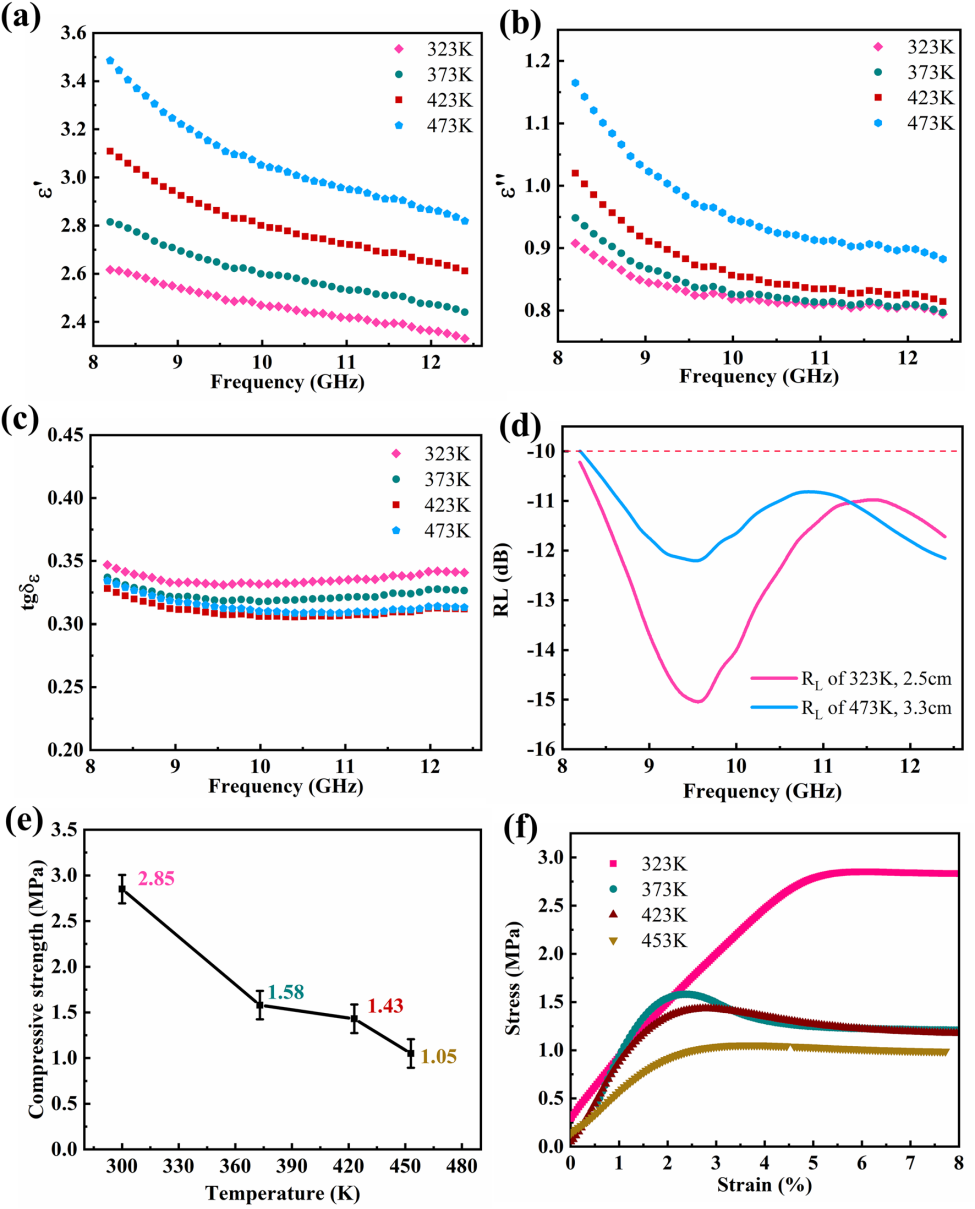
**a** Dielectric real part, **b** dielectric imaginary part, **c** dielectric loss tangent value, **d** *R*_*L*_ curves at different temperature, **e** compressive strength and **f** typical stress–strain curves of CP-4 with different temperatures

Aside from the ultra-wideband absorption, exhibiting excellent absorbing properties are required for absorbing materials when encountering high-temperature harsh environments. The changes in dielectric parameters of the CP-4 composite at different temperatures are shown in Fig. 6a–b. As the temperature increases, the curve shape of the real and imaginary dielectric parts remains substantially unchanged, but the curve position is shifted upward. The tangent value of dielectric loss changes minimally on the whole and shows a slight trend of decreasing (Fig. 6c). With the increase in temperature, the dielectric loss ability gradually decreases, and the absorbing ability of composite materials also presents a trend of declining. The variation in dielectric parameters can be explained by the following formula in Debye relaxation theory:

8 $$ \varepsilon ^{\prime} = \varepsilon_{\infty } + \frac{{\varepsilon_{s} + \varepsilon_{\infty } }}{{1 + \omega^{2} \tau^{2} }} $$

9 $$  \varepsilon = \frac{{\varepsilon_{s} - \varepsilon_{\infty } }}{{1 + \omega^{2} \tau^{2} }}\omega \tau + \frac{\sigma }{{2\pi f\varepsilon_{0} }} $$

When the temperature of the absorbing material changes, the greatest influence is the time corresponding to the polarization relaxation. According to Eqs. (8) and (9), with the increase in temperature, the thermal kinetic energy of the activated molecule increases, and the faster the process tends to steady state, the shorter the relaxation time is. Therefore, the dielectric real part increases with the increase in temperature. Similarly, the temperature is positively correlated with conductivity. The increase in temperature will activate substantial free electrons, resulting in an increase in electrical conductivity, and an increase in the value of the dielectric imaginary part. According to Eq. (1), *R*_L_ values under different thicknesses are calculated. The thickness of composite material that can cover the whole X band increases with the increase in temperature. As the temperature increases from 323 to 473 K, the corresponding thickness increases from 2.5 to 3.3 cm, which suggests a slight decrease in absorption performance.

If the absorbing material has both absorbing function and excellent mechanical properties, it can play a role in the integration of structure and function in aerospace applications, so that more space can be saved for other functions. A rigid foam is generally formed by co-curing process to prepare a sandwich structure. High temperature and pressure are required in the curing process. The maximum curing temperature can reach 453 K, and the curing pressure can reach 0.6 MPa. With the increase in temperature from 300 to 453 K, the compressive strength of the CP-4 composite decreases from 2.85 to 1.05 MPa (Fig. 6d). At 453 K, the compressive strength retention ratio of CP-4 is 36.8%, which is still higher than that at 0.6 MPa. The imide ring structure endows the composite with rigidity, so that it can maintain dimensional stability during curing process. At the same time, in engineering applications, CP-4 composite materials can also play the role of absorbing wave and bearing pressure in high-temperature and harsh environments. Typical stress–strain curves show that CP-4 exhibits good toughness (Fig. 6e). After yielding, CP-4 has no brittle fracture, instead of maintaining a high compressive capacity in a long strain range. In engineering applications, this feature provides a guarantee for structural security.

## Conclusion

In conclusion, CP composites with different CF contents were prepared by free radical copolymerization. The MA properties of the CP composites were tested through experiments and simulations to verify the feasibility of the porous structure to broaden the EAB. Notably, by constructing a porous structure, the EAB of the CP composites can reach an excellent ultra-wideband of 14 GHz in the range of 2–18 GHz. Meanwhile, the compressive strength of the CP composites is 1.05 MPa at 453 K, which fully meets the conditions for preparing sandwich structures in aeronautics and astronautics engineering applications. Moreover, the CP composites can still cover the X band (8.2–12.4 GHz) commonly used in military stealth at 473 K. They meet the requirements of lightweight and high strength in the field of ultra-wide frequency absorption and high-temperature environments.

## Supplementary Information

Below is the link to the electronic supplementary material.

Supplementary file1 (PDF 980 kb)
