# Supplementary material for: Achieving Ultra-Wideband and Elevated Temperature Electromagnetic Wave Absorption via Constructing Lightweight Porous Rigid Structure
Source: Nanomicro Lett. 2022 Aug 23;14:173. doi: 10.1007/s40820-022-00904-7 (PMC9399338; doi:10.1007/s40820-022-00904-7)
Supplement: Supplementary file 1 — Supplementary file1 (PDF 980 kb) [file 40820_2022_904_MOESM1_ESM.pdf]

Supporting Information for

Achieving Ultra-Wideband and Elevated Temperature Electromagnetic Wave Absorption via Constructing Lightweight Porous Rigid Structure

Zibao Jiao[1, 2], Wenjun Huyan[1, 2], Feng Yang[1, 2], Junru Yao[1, 2], Ruiyang Tan[3], Ping Chen[3], Xuewei Tao[4], Zhengjun Yao[1, 2, *], Jintang Zhou [1, 2, *], Peijiang Liu[1, *]

[1] College of Materials Science and Technology, Nanjing University of Aeronautics and Astronautics, Nanjing 211100, People’s Republic of China

[2] Key Laboratory of Material Preparation and Protection for Harsh Environment (Nanjing University of Aeronautics and Astronautics), Ministry of Industry and Information Technology, Nanjing 211100, People’s Republic of China

[3] School of Electronic Science and Engineering, Nanjing University, Nanjing 210023, People’s Republic of China

[4] School of Materials Science and Engineering, Nanjing Institute of Technology, Nanjing 211167, People’s Republic of China

* Corresponding authors. E-mail: yaozj@nuaa.edu.cn (Zhengjun Yao), imzjt@nuaa.edu.cn (Jintang Zhou), liupeijiang@ceprei.com (Peijiang Liu)

Supplementary Figures

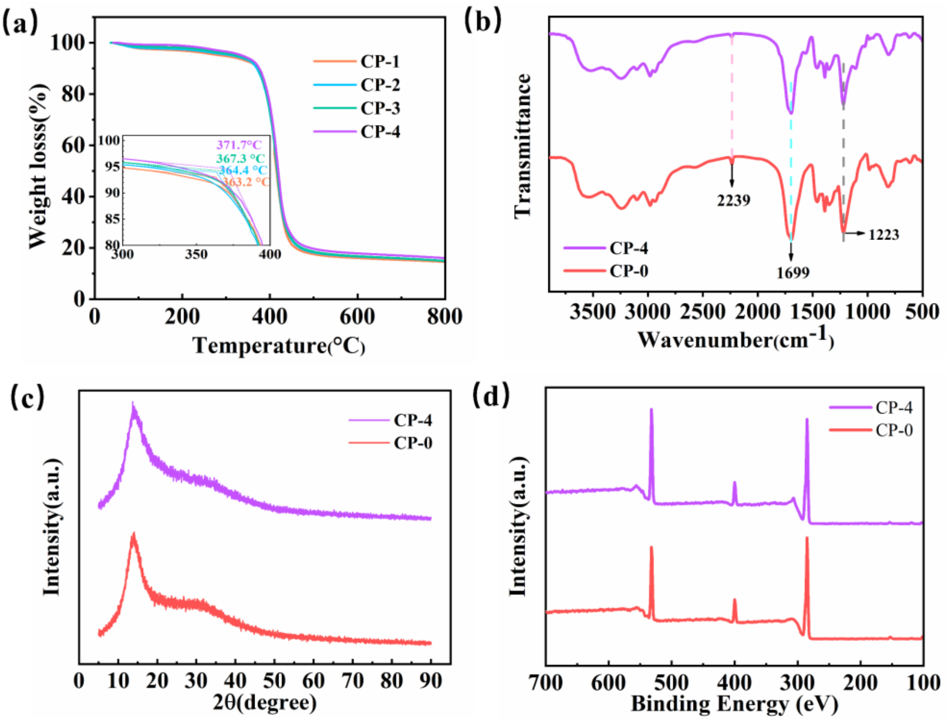

Fig. S1 (**a**) TG curve, (**b**) infrared spectrum, (**c**) XRD and (**d**) XPS of the samples

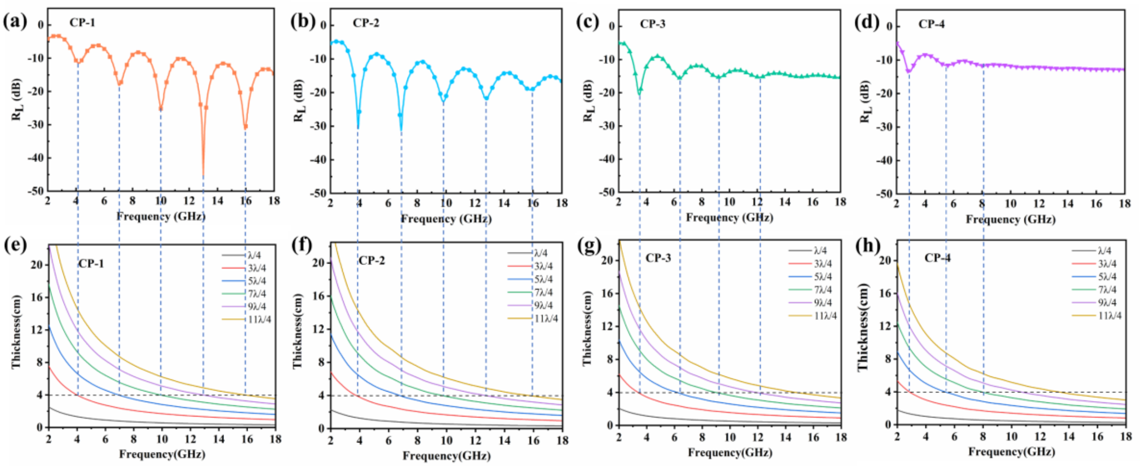

Fig. S2 R_L curve of (**a**) CP-1, (**b**) CP-2, (**c**) CP-3, (**d**) CP-4 with the thickness of 4 cm, λ/4 curve of (**e**) CP-1, (**f**) CP-2, (**g**) CP-3 and (**h**) CP-4

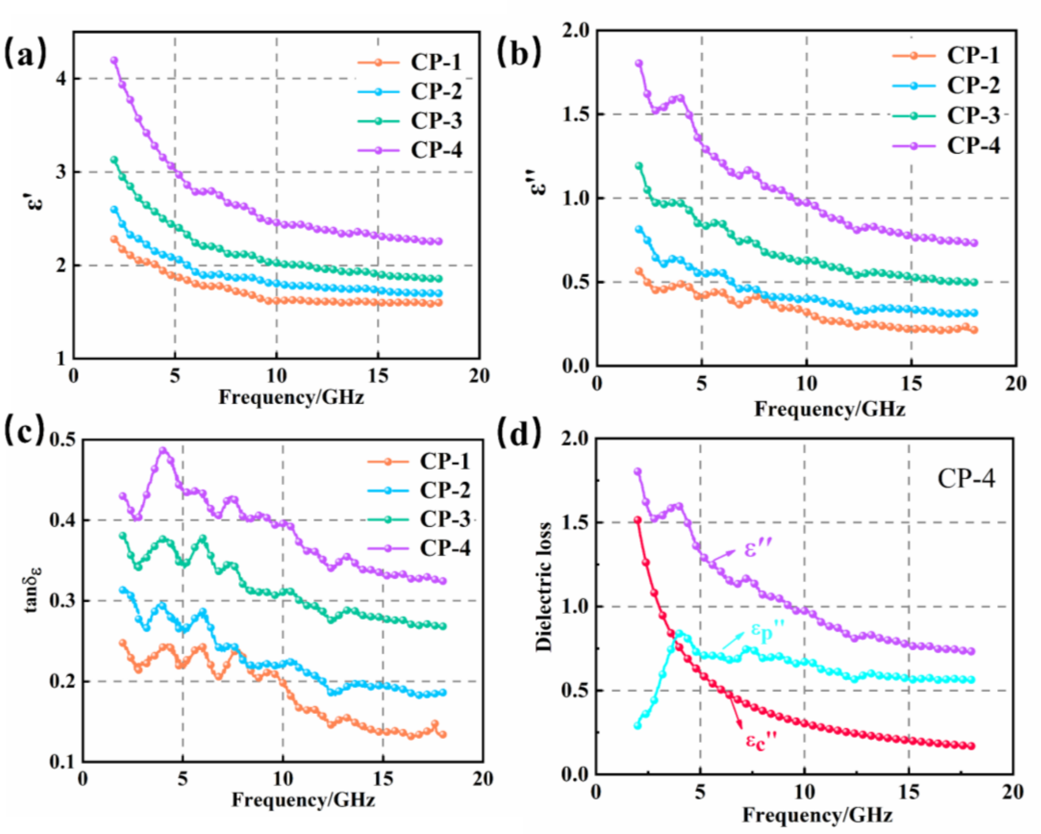

Fig. S3 (**a**) Real part ε′, (**b**) imaginary part ε″, (**c**) dielectric loss tangent and (**d**) polarization loss and conductive loss of the samples

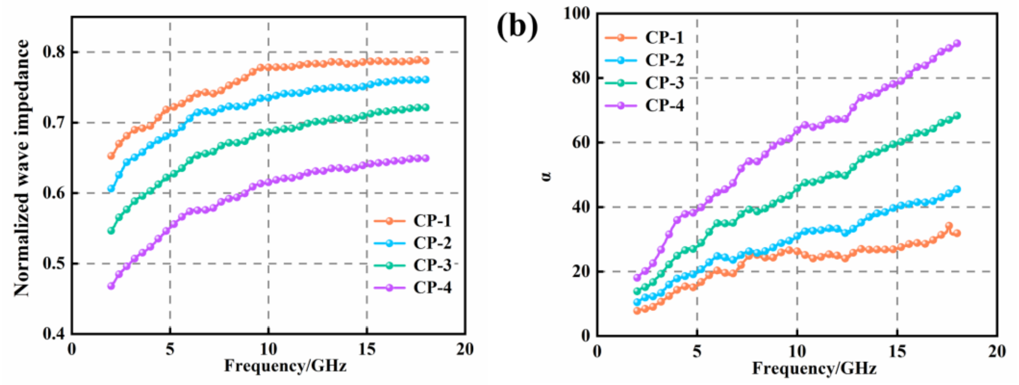

Fig. S4 (**a**)Normalized wave impedance and (**b**) attenuation coefficient α of the samples

For the influence of foaming ratio and bubble size, two groups of experiments were conducted: using the same amount of carbon fiber, foaming was carried out at different temperatures to obtain foam with different foaming rate. With CP-4 composite as the control sample, the foaming temperature was increased to 245 and 250 ℃ respectively with the same carbon fiber content, and labeled as CP-5 and CP-6. The density of polymer board before foaming is 1.15 g cm^{-3}, and the density of foam material after foaming is 110 mg cm^{-3}, 94 mg/cm^3 and 80 mg cm^{-3} respectively. The calculated foaming ratio is 10.5, 12.2 and 14.4 times respectively. As can be seen from **Fig. S5**, with the increase of foaming ratio, the size of the bubble hole gradually increases. Under this situation, the volume percentage of CF gradually declines with the increase of foaming rate under the same mass content. Therefore, the foams exhibit a gradual declining trend in dielectric parameters as well as the corresponding microwave absorption capability. (**Fig. S5**f).

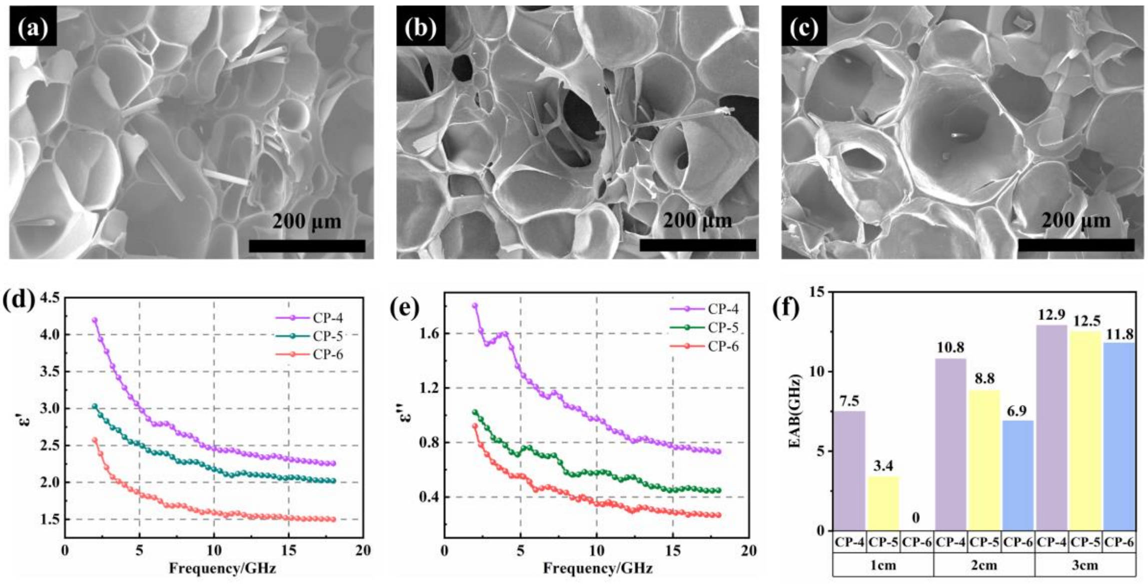

Fig. S5 SEM of(**a**)CP-4, (**b**) CP-5, (**c**) CP-6, (**d**) ε’, (**e**) ε” and (**f**) EAB with different thickness of the samples

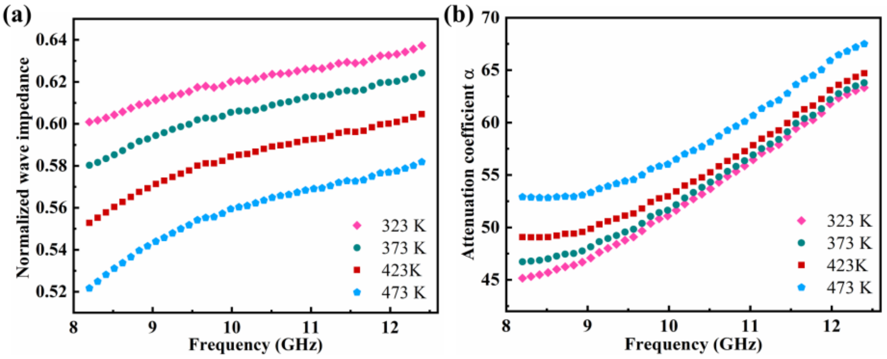

Fig. S6 (**a**)Normalized wave impedance and (**b**) attenuation coefficient α of CP-4 with different temperatures
